# IGF-I and Hyaluronic Acid Mitigate the Negative Effect of Irradiation on Human Skin Keratinocytes

**DOI:** 10.3390/cancers14030588

**Published:** 2022-01-24

**Authors:** Celena A. Sörgel, Rafael Schmid, Nina Stadelmann, Volker Weisbach, Luitpold Distel, Raymund E. Horch, Annika Kengelbach-Weigand

**Affiliations:** 1 Laboratory for Tissue Engineering and Regenerative Medicine, Department of Plastic and Hand Surgery, University Hospital of Erlangen, Friedrich-Alexander University Erlangen-Nürnberg (FAU), 91054 Erlangen, Germany; celena.soergel@uk-erlangen.de (C.A.S.); rafael.schmid@uk-erlangen.de (R.S.); nina.stadelmann@extern-uk-erlangen.de (N.S.); Annika.Kengelbach-Weigand@uk-erlangen.de (A.K.-W.); 2 Department of Transfusion Medicine and Haemostaseology, University Hospital of Erlangen, Friedrich-Alexander University Erlangen-Nürnberg (FAU), 91054 Erlangen, Germany; Volker.Weisbach@uk-erlangen.de; 3 Department of Radiation Oncology, University Hospital of Erlangen, Friedrich-Alexander University Erlangen-Nürnberg (FAU), 91054 Erlangen, Germany; luitpold.distel@uk-erlangen.de; 4 Comprehensive Cancer Center Erlangen-EMN (CCC ER-EMN), 91054 Erlangen, Germany

**Keywords:** keratinocytes, irradiation, hyaluronic acid, IGF-I, dermatitis, wound healing

## Abstract

**Simple Summary:**

Patients undergoing radiation therapy for the treatment of various types of cancer often experience side effects such as radiation dermatitis. A gold standard treatment is still lacking. The objective of the present study was to find novel therapeutic strategies for the regeneration and repair of damaged skin areas after irradiation. An in vitro 2D and 3D primary keratinocyte model was used to test the effect of insulin-like growth factor I (IGF-I), keratinocyte growth factor (KGF), platelet lysate (PL), hyaluronic acid (HA), and adipose-derived stem cell (ADSC) conditioned medium on the functional abilities (viability, migration) and the gene expression of irradiated keratinocytes. Hyaluronic acid and IGF-I effectively reduced the irradiation damage of primary keratinocytes by stimulating viability and migration and reducing cell apoptosis and necrosis. These findings indicate that the negative effects of irradiation on keratinocytes located in the patient’s skin can be counterbalanced with HA and IGF-I treatment.

**Abstract:**

Ionizing radiation has become an integral part of modern cancer therapy regimens. Various side effects, such as radiation dermatitis, affect patients in acute and chronic forms and decrease therapy compliance significantly. In this study, primary keratinocytes were irradiated in a 2-dimensional (2D) culture as well as on a 3-dimensional (3D) collagen-elastin matrix with doses of 2 and 5 Gy. The effect of different concentrations of IGF-I, KGF, platelet lysate (PL), high and low molecular weight hyaluronic acid (H-HA, L-HA), and adipose-derived stem cell (ADSC) conditioned medium was analyzed in respect to cell viability (WST-8), wound closure (migration), and the gene expression (quantitative real-time PCR) of 2D cultures. The 3D culture was evaluated by WST-8. A mixture of H-HA and L-HA, as well as IGF-I, could significantly stimulate the keratinocyte viability and migration which were severely reduced by irradiation. The MKI67and IL6 gene expression of irradiated keratinocytes was significantly higher after H-HA/L-HA treatment. The stimulating effects of H-HA/L-HA and IGF-I were able to be confirmed in 3D culture. A positive influence on cell viability, migration, and gene expression was achieved after the treatment with H-L-HA and IGF-I. These results open the possibility of a novel therapeutic method for both the prevention and the treatment of radiation dermatitis.

## 1. Introduction

Keratinocytes are part of the outermost protection layer of the human body. The protective keratinocyte layer, however, can be affected as an accidental or inevitable consequence of external influences. Ionizing radiation has become an integral part of modern cancer therapy regimens as an adjuvant or a neoadjuvant approach. In general, a possible pathophysiological negative effect on the skin may occur during the course of irradiation therapy [1]. Radiation resembles the standard treatment protocol for certain cancer types, such as head and neck, breast, and lung cancers [2]. Even though it represents a crucial part of the treatment protocol for malignant diseases, it can have many adverse effects, ranging from acute toxicity, such as acute radiation syndrome, to chronic sequelae related to stimulation of the inflammatory and necrotic processes that may necessitate secondary measures [1,3,4,5,6]. Radiation can damage human tissue—either by direct or indirect pathways—leading to a disruption of the physiological healing process which normally comprises hemostasis, inflammation, proliferation, and skin remodeling [7]. The disruption of this process causes cell death of the irradiated tumor tissue as well as possible damage to the surrounding tissue [8,9,10].

The extent of the damage varies and depends on several general factors: the radiation dose, the patient’s individual radiosensitivity due to their genetics and immune system, the status of the connective tissue, which can be affected by rheumatologic disorders such as lupus or scleroderma as well as specific factors such as the state of the cell cycle checkpoint activators [11,12,13,14]. In addition, different body areas show variable reactions to radiotherapy, depending on the skin thickness and the degree of area exposure. Areas with thinner skin are involved more severely [15]. Patient symptoms range from transient hyperemia or erythema (<30 days), induced by lower irradiation doses, to dermal ischemia, atrophy, desquamation, fibrosis, and necrosis induced by microvascular damage. These can occur after irradiation doses > 10 Gy [16]. This condition can lead to chronic wounds, possible superinfections, and deep tissue erosions as long-term side effects [17].

The standard treatment for irradiation side effects includes topical regimens, e.g., normal washing and personal hygiene, emollient creams, topical steroid or nonsteroidal creams, and silver dressings. Further systemic interventions with intravenous amifostine or pentoxifylline or oral enzymes may sometimes be needed [18]. Modifications in the irradiation technique, such as intensity-modulated radiotherapy, can be used as a preventive measure [19,20,21]. It has been proposed that tissue-engineered skin substitutes could enhance wound healing after radiation therapy [22]. Despite having clearly established treatment protocols, patients may still suffer from acute pain and radiation dermatitis during the treatment period, as well as from systemic side effects, such as mobility and sleep problems. Therefore, there is an urgent need for novel treatment options leading to faster recovery and reduction of the suffering of patients with radiation damage [23,24]. The abovementioned side effects still prevail and affect 95% of all patients undergoing radiation therapy [11,17,25]. A customized wound dressing containing primary keratinocytes would be an ideal therapeutic strategy for the regeneration and repair of radiation-damaged skin areas. This dressing should ideally contain an ingredient combination focused on the greatest possible reduction in radiation damage. We have chosen growth factors with a positive influence on keratinocyte survival. As a first step toward this direction, we evaluated their effects on the keratinocytes’ functional abilities. In vitro 2D and 3D primary keratinocyte models were used to test the effect of insulin-like growth factor I (IGF-I), keratinocyte growth factor (KGF), platelet lysate (PL), hyaluronic acid (HA), and adipose-derived stem cell (ADSC) conditioned medium. The viability, migration, and gene expression of irradiated keratinocytes were evaluated to exemplify cell repopulation and wound closure time and to demonstrate the epigenetic changes. The results of this study could be used as a basis for potential new treatment options. It could promote earlier wound healing and complete regeneration of the skin and even be used as a prevention strategy to completely avoid the chronic and acute sequelae resulting from irradiation.

## 2. Materials and Methods

### 2.1. Patients

Human tissue for the isolation of primary cells was collected from patients undergoing abdominoplastic surgery after weight loss. The tissue collection was approved by the Friedrich-Alexander University Erlangen-Nürnberg (FAU) (Germany, Ethics number 264_13B, 14.11.2013; 424_18B 19.11.2018) in conformity with the university’s ethics commission and the World Medical Association Declaration of Helsinki. Two primary cell isolates were used. Keratinocytes isolated from a 42-year-old female patient were used for the 2D experiments. The cells isolated from a 41-year-old healthy female patient were used for the 3D experiment. Written informed consent was obtained from both patients. 

### 2.2. Isolation of Primary Keratinocytes

For the isolation procedure of the keratinocytes, the human Epidermis Dissociation Kit (Miltenyi Biotec B.V. & Co. KG, Bergisch-Gladbach, Germany), including Enzyme G, Enzyme P, and Enzyme A with the addition of RPMI 1640 (Thermo Fisher Scientific, Waltham, MA, USA), was used. The subcutaneous fat was removed from the tissue and the skin was cut into pieces of approximately 4 mm in diameter. The samples were incubated for 18 h at 4 °C in a mixture of RPMI 1640 and Enzyme G. Afterwards, the epidermis was peeled off from the dermis. The epidermal layers were incubated in Enzyme P and Enzyme A at 37 °C for 60 min. The epidermis was transferred into the gentleMACS™ C Tube and placed on the gentleMACS™ Octo Dissociator (Miltenyi Biotec B.V. & Co. KG). Program B was chosen. The samples were filtered (70 µm pore size), centrifuged (300× *g*, 10 min), and resuspended in Keratinocyte Growth Medium 2 (KGM 2) (PromoCell GmbH, Heidelberg, Germany). The KGM 2 included a supplement mix consisting of CaCl2 (0.06 mM), 10 µg/mL recombinant human transferrin-5, 0.33 µg/mL hydrocortisone-165, 0.39 µg/mL epinephrine-195, 0.004 mL/mL bovine pituitary extract, 0.125 ng/mL recombinant human epidermal growth factor, 5 µL/mL recombinant human insulin, and 1% penicillin-streptomycin (100 U/mL) (Sigma-Aldrich Corporation, St. Louis, MO, USA). The keratinocytes were seeded in T75 cell culture flasks coated with 3.0 mg/cm^2^ rat-tail collagen type I (Sigma-Aldrich Corporation) and incubated at 37 °C, 5% CO_2_. The medium was changed every 48 h and the cells were split at 80–90% confluence. The keratinocytes were used up to passage 4.

### 2.3. Adipose-Derived Stem Cell Conditioned Medium

Immortalized adipose-derived stem cells (ADSC), ASC/TERT1, from Everycyte (Everycyte GmbH, Vienna, Austria), were cultivated in an Endothelial Cell Basal Medium-2 (EBM^®^-2) (Clonetics^®^, Lonza Group AG, Basel, Switzerland) enriched with the supplement mix, 2% FCS superior (Sigma-Aldrich Corporation), and 200 µg/mL geneticin (Sigma-Aldrich Cooperation) up to a confluence of 80–90%. The cell layers were washed twice with phosphate-buffered saline (PBS) (Sigma Aldrich Corporation) and incubated in EBM^®^-2 (Clonetics^®^, Lonza Group AG) without the supplement mix for 24 h at 37 °C and 5% CO_2_. The conditioned medium (CM) was concentrated using centrifugal cellulose filter tubes (Amicon^®^ Ultra 15, Merck Millipore, Burlington, MA, USA). After centrifugation at 4000× *g* for 30 min, the supernatant was diluted with keratinocyte basal medium 2 (KBM 2) (PromoCell GmbH) supplemented with CaCl2 (0.06 mM), 10 µg/mL recombinant human transferrin-5, 0.33 µg/mL hydrocortisone-165, and 0.39 µg/mL epinephrine-195 (from the Keratinocyte Growth Medium 2 KIT) to obtain 3-fold CM. In parallel, a control group was prepared by incubating the EBM without FCS without the cells for 24 h at 37 °C and 5% CO_2_. The concentration was carried out in the same manner as for the ADSC CM.

### 2.4. Production of Platelet Lysate

The PL was collected at the Department of Transfusion Medicine and Haemostaseology, University Hospital of Erlangen, Germany, from blood donations from healthy volunteers by plateletpheresis. After pooling, the samples were stored at −80 °C until further usage.

### 2.5. Cell X-ray Irradiation

Irradiation of the keratinocytes in 2D and on MatriDerm^®^ scaffolds was performed with X-rays at a voltage of 120 kV and a 2 mm aluminum filter using an Isovolt Titan 160 X-ray tube (GE Sensing & Inspection Technologies, Ahrensburg, Germany). Irradiation doses of 2 Gy or 5 Gy were achieved at a dose rate of 4 Gy per minute, and the focus-field distance was 21 cm. 

### 2.6. Irradiation of Keratinocyte 2D Cell Cultures

The keratinocytes were seeded in triplicates at a density of 1.5 × 10^3^ cells/well in 100 µL KGM 2 in 96-well plates coated with 3.0 mg/cm^2^ rat-tail collagen type I. After 24 h, the cells were irradiated with doses of 2 Gy or 5 Gy. A control group was not irradiated. The medium was changed one hour after irradiation to KBM 2 implemented with CaCl2 (0.06 mM), 10 µg/mL recombinant human transferrin-5, 0.33 µg/mL hydrocortisone-165, and 0.39 µg/mL epinephrine-195 (all supplements were from Supplement Pack KGM 2). According to the experimental groups, specific supplements were added to the medium (Table 1).

Cell viability was analyzed by a WST-8 assay (PromoCell GmbH) at time points 1, 4, and 7 days after irradiation. Ten µL CCVK1-reagent was added to each well and incubated for 2 h at 37 °C and 5% CO_2_. For each group, a blank sample was prepared containing the respective medium without cells. Absorbance was measured at 450 nm and 600 nm (background) (Multiscan Go and SkanIT RE for Multiscan Go 6.1, Thermo Fisher Scientific). Subsequently, the wells were washed with PBS, fixed with 4% formaldehyde (ROTI^®^Histofix, Carl Roth GmbH + Co. KG, Fürth, Germany), and stained with 1 µg/mL 4′,6-Diamidin-2-phenylindol (DAPI) (Life Technologies, Carlsbad, CA, USA). Microscopic images were taken at 40- and 100-fold magnification (Olympus IX83, CellSens software, Olympus Corporation, Shinjuku, Japan).

Cell migration was analyzed after irradiation by the Oris™ Cell Migration Assay (Platypus Technologies, Madison, WI, USA). For the creation of a cell-free area, cell seeding stoppers were introduced into the well of a collagen-coated (3.0 mg/cm^2^, rat-tail collagen type I, Sigma-Aldrich Corporation) 96-well plate. Subsequently, the keratinocytes were seeded in triplicates into the wells at a concentration of 6 × 10^4^ cells/well in 100 µL KBM 2 supplemented with CaCl2 (0.06 mM), 10 µg/mL recombinant human transferrin-5, 0.33 µg/mL hydrocortisone-165, and 0.39 µg/mL epinephrine-195 and incubated at 37 °C and 5% CO_2_ for 4 h. The cells were irradiated with 2 Gy or 5 Gy. A control group was not irradiated. After 4 h, the cell seeding stoppers were removed, the cells were washed with PBS, and the medium was changed according to the experimental groups (Table 1). Cell migration into the cell-free area was analyzed by microscopy at 40-fold magnification (1 central image/well) after 0 h, 8 h, and 16 h (Olympus IX83, cellSens software, Olympus Corporation, Tokyo, Japan). Analysis was conducted with the Fiji Is Just ImageJ (Fiji) 1.51u (extended distribution). The migration data were normalized to time point 0 h set as 1. 

### 2.7. RNA Isolation, Reverse Transcription, and Quantitative Real-Time PCR

The keratinocytes were seeded at a cell density of 1.5 × 10^5^ cells/flask in 5 mL KGM 2 in T25 cell culture flasks coated with 3.0 mg/cm² rat-tail collagen type I until a confluence level of 80% was reached. The cells were irradiated with 2 Gy or 5 Gy. A control group was not irradiated. One hour after irradiation, the medium was changed to KBM 2 supplemented with CaCl2 (0.06 mM), 10 µg/mL recombinant human transferrin-5, 0.33 µg/mL hydrocortisone-165, and 0.39 µg/mL epinephrine-195 (all supplements were from Supplement Pack KGM 2) and IGF-I (100 ng/mL) or H-L-HA (50/50) (1 mg/mL). After incubation for 24 h at 37 °C with 5% CO_2_, the samples were washed with PBS, detached from the cell culture flask and centrifuged at 300× *g* for 4 min. The resulting pellet was frozen in liquid nitrogen and stored at −80 °C. RNA was isolated using the RNeasy^®^Mini Kit (Qiagen GmbH, Hilden, Germany + QIAshredder), using the manufacturer’s recommendations. NanoDrop (Thermo Fisher Scientific) was used for measuring the RNA concentration. RNA (1 µg) was transcribed to cDNA with the QuantiTect^®^ Reverse Transcription Kit (Qiagen GmbH) according to the standard protocol. For the qRT-PCR, SsoAvanced™ Universal SYBR^®^ Green Supermix (Bio-Rad Laboratories Incorporation, Hercules, California USA) was used. The NCBI gene database was used for the primer design. GAPDH served as the housekeeping gene. All the primers (Table 2) were purchased from the Sigma-Aldrich Corporation.

### 2.8. Irradiation of Keratinocytes on a 3D Collagen-Elastin Matrix

The collagen-elastin matrix (MatriDerm^®^, Dr. Suwelack Skin and Health Care, Billerbeck, Germany) was cut into pieces with a 6 mm biopsy punch under sterile conditions. The keratinocytes were seeded in triplicates. The cells were suspended in 30 µL KGM 2 and gently applied onto the scaffold at a cell density of 5 × 10^5^ cells per scaffold. The scaffolds were transferred into 48-well-plates and covered with 300 µL KGM 2. After 24 h at 37 °C and 5% CO_2_, the scaffolds were irradiated with 2 Gy or 5 Gy. A control group was not irradiated. The scaffolds were treated with IGF-I (100 ng/mL) and H-HA/L-HA (1 mg/mL) 1 h after irradiation. A control group received the same medium without IGF-I or H-HA/L-HA 1 mg. Cell viability was analyzed by a WST-8 assay (PromoCell GmbH) at time points 1, 4, and 7 days after irradiation. Ten µL CCVK1-reagent was added to each well and incubated for 2 h at 37 °C and 5% CO_2_. For each group a blank sample was prepared containing the respective medium without cells. Absorbance was measured at 450 nm and 600 nm (background).

For microscopic analysis of the cell survival, the Apoptotic/Necrotic/Healthy Cells Detection Kit (Promokine, Heidelberg, Germany) was used according to the manufacturer’s recommendations. On days 1, 4, and 7, the cell layers were washed twice with the binding buffer solution. A staining solution was prepared by mixing 5 μL of FITC-Annexin V, 5 μL of Ethidium Homodimer III, and 5 μL of Hoechst 33342 to 100 μL of the binding buffer. The cells were incubated for 15 min at room temperature, washed with the buffer solution and immediately analyzed with the Olympus IX83 microscope using the FITC, TRITC, and Hoechst channels at 100-fold magnification.

### 2.9. Statistical Analysis

The data were statistically analyzed with Graph Pad Prism 7 (Graph Pad Software) using the Dunns’ and the Tukey Test. The data are presented as mean ± standard deviation. Statistical significance was defined by *p* values ≤ 0.05. For better visibility, the contrast and brightness of the images were adjusted by PowerPoint (Microsoft Cooperation, Redmond Washington, DC, USA).

## 3. Results

### 3.1. Cell Viability

**Figure 1:**
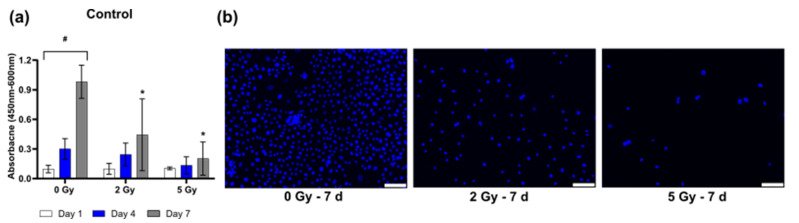
Keratinocyte viability after irradiation of the control groups without growth factor treatment: (**a**) Y–axis shows absorbance on 1 d, 4 d, and 7 d after 0, 2, 5 Gy irradiation; (**b**) representative images of irradiated keratinocytes without growth factor supplementation at time point 7 d stained with DAPI (blue = living cells). * *p* ≤ 0.05, significances were correlated to the non-irradiated respective control. * = significant compared to the control without growth factors in the respective irradiation dose at the respective time point. # = significances were correlated to the control on day 1. Scale bar 100 µm.

The cell viability was reviewed on 1 d, 4 d, and 7 d to analyze the influence of irradiation on the keratinocyte viability. All the control groups, 0 Gy, 2 Gy, and 5 Gy without addition of growth factors, showed an increase in cell viability over time (Figure 1a).

The irradiation induced a significant decrease in cell viability after 7 d compared to the non-irradiated group (Figure 1a). Through DAPI staining, it was shown that not only the cell viability but also the cell proliferation was reduced after irradiation (Figure 1b).

**Figure 2:**
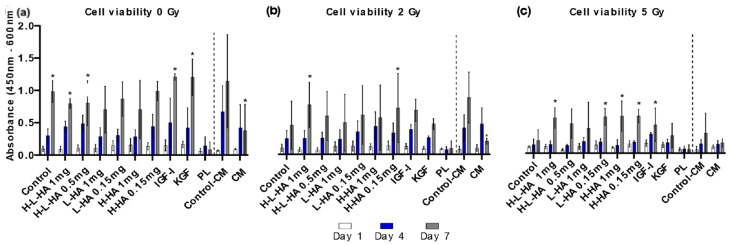

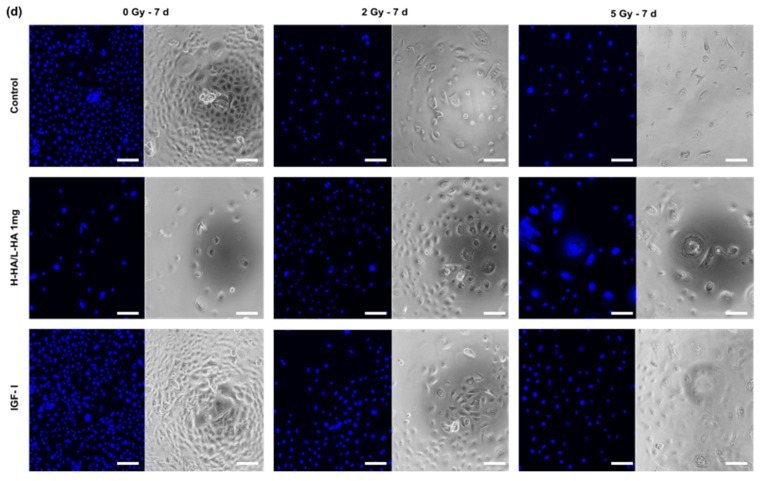
Keratinocyte viability of the irradiation groups, 0, 2, and 5 Gy with growth factor treatment, on 1 d, 4 d, and 7 d: (**a**) cell viability without irradiation; (**b**) cell viability after irradiation with 5 Gy; (**c**) cell viability after irradiation with 2 Gy; (**d**) representative images of irradiated keratinocytes at time point 7 d stained with DAPI (blue = living cells) and PH. Scale bar 200 µm. * *p* ≤ 0.05, significances were correlated to the respective control. Scale bar = 200 µm.

In all the experimental groups, except 50 µL/mL PL, the viability increased from 1 d to 7 d (Figure 2a–c). From 1 d to 7 d, the non-irradiated control cells without growth factor treatment showed an 8.8-fold increase in viability, whereas the cell viability of the 2 Gy and 5 Gy irradiated control cells increased 4.8-fold and 1.9-fold, respectively. One mg/mL H-HA/L-HA, 0.15 mg/mL L-HA, 0.15 mg/mL H-HA, 1 mg/mL H-HA, and 100 ng/mL IGF-I induced a trend towards an increased cell viability after 7 days in the 5 Gy group compared to the cells without growth factor supplementation on day 1 (Figure 2a–c). Likewise, a higher number of DAPI-stained cells were detected in these groups, indicating a higher degree of cell proliferation (Figure 2d). There was no stimulating effect of 50 µL/mL PL or 3-fold concentrated ADSC-CM on the keratinocyte viability in any group. The cell viability was always lower compared to the respective control group (Figure 2a–c). 

### 3.2. In Vitro Wound Closure Time

**Figure 3:**
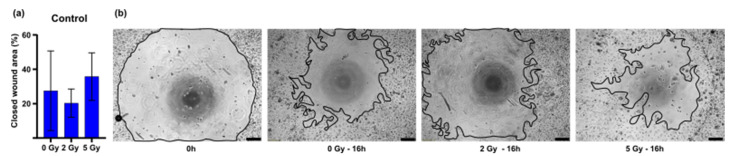
Keratinocyte migration of the irradiated control groups without growth factor treatment: (**a**) Y-axis shows closed wound area at time point 16 h after 0, 2, 5 Gy irradiation; (**b**) representative images of irradiated keratinocytes at time point 0 h and 16 h. Scale bar 200 µm.

The cell migration was measured microscopically after 16 h to analyze the influence of the irradiation. Keratinocyte migration was visible in every group. The irradiation had no significant effect on the migration behavior of the keratinocytes. We observed a tendency of increased migration after irradiation with 5 Gy (Figure 3).

**Figure 4:**
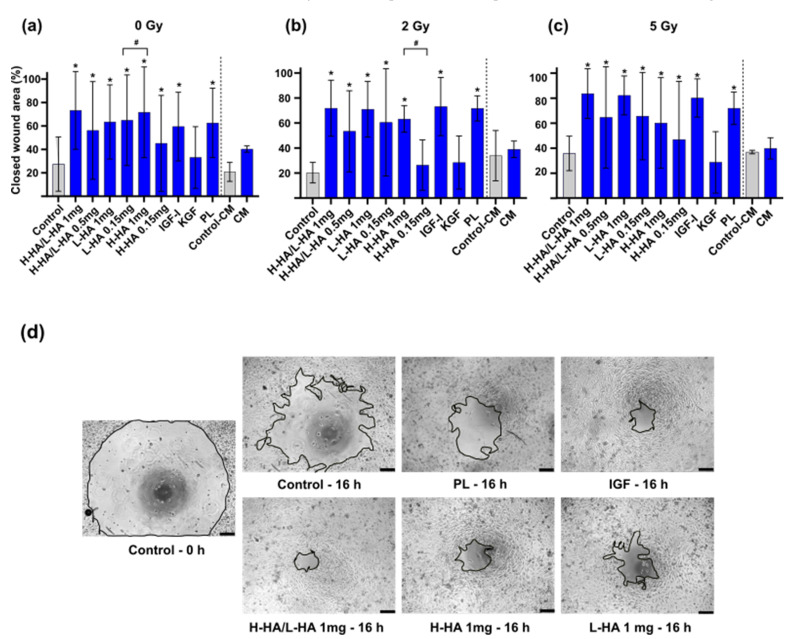
Keratinocyte migration of the irradiation groups 0, 2, and 5 Gy with growth factors after 16 h: (**a**) Y-axis shows closed wound area without irradiation; (**b**) Y-axis shows closed wound area after 2 Gy irradiation; (**c**) Y-axis shows closed wound area after 5 Gy irradiation; (**d**) representative images of irradiated keratinocytes at time point 0 h and 16 h. *p* ≤ 0.05, significances were correlated to the respective control. * = significant compared to the control without growth factors in the respective irradiation dose at the respective time point. # = significant compared to the lower dose of HA. Scale bar 200 µm.

In the non-irradiated control group, as well as in both irradiated groups, there was a distinct increase in migration after treatment with 1 mg/mL H-HA/L-HA, 1 mg/mL L-HA, 1 mg/mL H-HA, 100 ng/mL IGF-I, and 50 µL/mL PL compared to the control group. This was also clearly observed in the phase contrast images (Figure 4a–d). In the 5 Gy group, 1 mg/mL H-HA/L-HA, 1 mg/mL L-HA, 100 ng/mL IGF-I, and 50 µL/mL PL induced a 2-fold migration rate compared to the control (Figure 4c). The higher-concentrated H-HA induced a significant higher migration in the non-irradiated control group and after irradiation with 2 Gy when compared to the respective lower concentrations (Figure 4a,b).

### 3.3. Gene Expression (qPCR)

**Figure 5:**
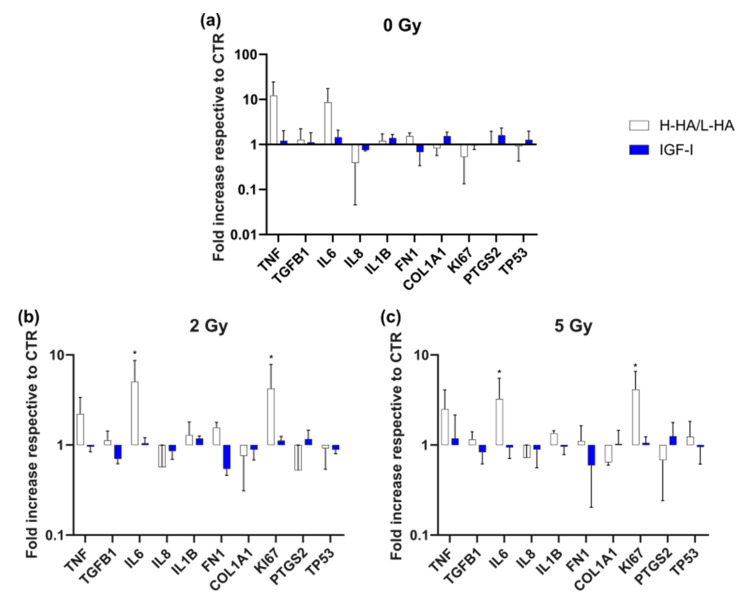
Gene expression of keratinocytes of irradiation groups, 0, 2, and 5 Gy, after 1 mg/mL H-HA/L-HA and 100 ng/mL IGF-I treatment after 24 h. Y-axis shows fold increase respective to control (CTR) (set to 1). (**a**) Gene expression without irradiation; (**b**) with 2 Gy irradiation; (**c**) with 5 Gy irradiation. * *p* ≤ 0.05, significances were correlated to the respective control. * = significant compared to the control without growth factors in the respective irradiation dose at the respective time point.

The gene expression of the irradiated cells was analyzed 24 h after treatment with 1 mg/mL H-HA/L-HA or 100 ng/mL IGF-I (Figure 5). There was a higher expression of TNF and a clearly significant higher expression of IL6 and MKI67 after treatment with 1 mg/mL H-HA/L-HA compared to the control in both irradiation groups (7.13- and 5.76-fold after 2 Gy irradiation, 4.40- and 5.50-fold after 5 Gy irradiation) (Figure 5b,c).

### 3.4. Cell Viability on the 3D Collagen-Elastin Scaffold

**Figure 6:**
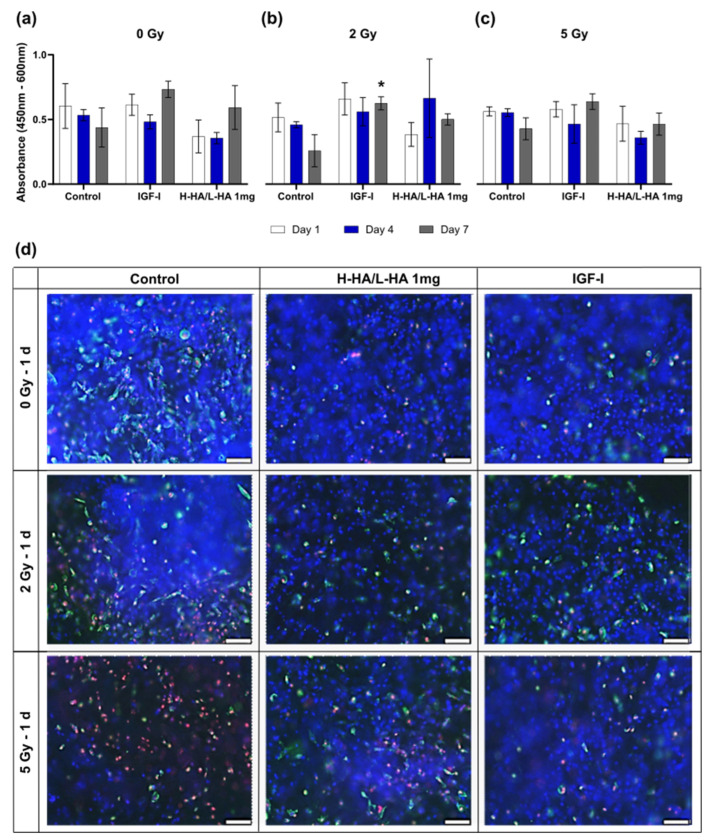
Keratinocyte viability on 3D MatriDerm^®^ collagen-elastin scaffolds of the irradiation groups 0, 2, and 5 Gy with 1 mg/mL H-HA/L-HA and 100 ng/mL IGF-I supplementation on 1 d, 4 d, and 7 d: (**a**) cell viability without irradiation; (**b**) cell viability after irradiation with 2 Gy; (**c**) cell viability after irradiation with 5 Gy; (**d**) representative images of irradiated keratinocytes at time point 1 d (living = blue, apoptotic = green, dead = red). * *p* ≤ 0.05, significances were correlated to the respective control. * = significant compared to the control without growth factors in the respective irradiation dose at the respective time point. Scale bar 100 µm.

The cell viability of the collagen-elastin scaffolds was measured on 1 d, 4 d, and 7 d. The cell viability of the irradiated cells treated with 1 mg H-HA/L-HA and 100 ng/mL IGF-I increased from 1 d to 7 d (Figure 6). The 100 ng/mL IGF-I stimulated cell viability caused a significant increase after 7 d in the 2 Gy group compared to the control. In the other groups, a trend of a higher cell viability could be observed, especially after 7 d in the non-irradiated and 2 Gy irradiated groups with 1 mg H-HA/L-HA and 100 ng/mL IGF-I, as well as in the 5 Gy group treated with 100 ng/mL IGF-I. After irradiation with 5 Gy, the viability of the untreated scaffold was equal to the controls but increased compared to the results after irradiation with 2 Gy. (Figure 6a–c).

Live-dead-apoptotic staining revealed an increase in dead and apoptotic cells after irradiation. When treated with 1 mg H-HA/L-HA and 100 ng/mL IGF-I, a higher number of living cells and a lower amount of dead apoptotic cells were visible in the 2 Gy and 5 Gy groups (Figure 6d).

## 4. Discussion

There still is a strong need for further research on the therapies for dermal complications induced by radiation therapy [15,23]. Within this study, the effect of different treatment options on irradiated keratinocytes was evaluated in a 2D and 3D in vitro culture model. It was the overall aim of this study to decipher possible therapeutic options for the complex pattern of direct tissue injury and inflammatory cell recruitment caused by skin irradiation. This involves, e.g., damage to epidermal basal cells, endothelial cells, and vascular components, as well as a reduction in Langerhans cells [17]. Thereby, skin exposure to ionizing radiation affects the normal wound-healing process [2,9,22]. While in human skin models multiple skin biopsies are necessary to study the effect of irradiation on the keratinocytes, in vitro skin cultures allow subsequent observations and standardized conditions [26].

In this study, we were able to demonstrate the well-known decrease in cell viability after irradiation. These findings are in concordance with the study of Tureson et al. [5,9,26]. With daily 1.1 Gy dose fractions, a continued and increasing cell depletion was observed in the skin. In severe radiation dermatitis, there is massive neutrophilic infiltration of the epidermis and profound apoptosis causing consequent cell death. With each dose of radiation, the opportunity for tissue healing due to cellular repopulation is reduced. Chronic radiation-induced changes in the skin are characterized by the disappearance of follicular structures, an increase in collagen, damage to the elastic fibers in the dermis, and a fragile epidermal covering [27]. This hypothesis underlining the damage to the skin is in line with the decrease in viability and functionality in our study results in the non-treated controls. 

On day 7, cell viability was significantly lower in the 2 Gy and 5 Gy irradiated group compared to the non-irradiated control. Interestingly, this effect could be significantly reversed when the keratinocytes were treated with 1 mg/mL H-HA/L-HA, 0.15 mg/mL L-HA, 0.15 mg/mL H-HA, 1 mg/mL H-HA, or 100 ng/mL IGF-I after irradiation with 5 Gy. From this, it can be concluded that irradiation damage to keratinocytes can be decreased and even fully compensated with HA or IGF-I. The general stimulating effect of IGF-I and HA on cell viability was described in previous studies; however, to the best of our knowledge, this is the first time their positive influence on gamma-irradiated damaged keratinocytes has been described. Sadagurski et al. demonstrated that human insulin-like growth factor 1 receptor (IGF-IR) signaling correlates positively with an increase in keratinocyte proliferation and a lower rate of cell differentiation [28]. A further study showed a positive correlation between IGF-IR signaling and increased cell survival after ultraviolet-B (UVB) irradiation [29]. Hasova et al. treated UVB-irradiated immortalized HaCaT with HA and demonstrated a decrease in the release of proinflammatory cytokines and an increase in cell viability [30]. The efficacy of IGF-I and HA in wound healing was also shown in vivo. IGF-I promotes in vivo wound healing and re-epithelialization in the mouse model by activating estrogen receptors [31]. These findings are in concordance with the study of Wang et al. [32]. After GMC3S depletion, an increase in IGF-IR was observed. This consequently led to significant improvements in the wound healing of diabetic mice [32]. Other studies showed improved in vivo wound healing and an increased protection against oxidative stress after application of HA scaffolds fortified with arginine derivates in the rat model [33,34]. Gelatin-hyaluronic acid-chondroitin scaffolds led to increased levels of the expression of wound-healing markers such as TGF-β1 and a reduction in the TNF-α and IL-6 expression in the swine model [35]. HA and IGF-I are both proven to have a positive influence on in vivo wound healing in various pathologic settings [31,32,33,34,35]. Considering the significant stimulating in vitro effect on irradiated keratinocytes we demonstrated in our study, positive effects on cell survival, repopulation, and wound closure time also can be anticipated in vivo. 

Within this study, the keratinocytes were further cultivated on a 3D elastin-collagen matrix for better mimicking of the in vivo situation. MatriDerm^®^ was chosen as a matrix because of its similarity to human skin, the successful keratinocyte culturing, and its in vivo applicability [28]. Similar to our 2D results, and underlining the high potential of HA and IGF-I, there was a significant stimulating effect by 100 ng/mL IGF-I and an increase in cell viability with 1 mg H-HA/L-HA after irradiation with 2 Gy, although it was not significant.

We further analyzed the migration of the irradiated cells under the influence of different growth factors as an important step for wound-healing capacity. In accordance with the WST-8 results, 1 mg/mL H-HA/L-HA and 100 ng/mL IGF-I significantly stimulated irradiated and non-irradiated keratinocyte migration. These results are in line with those reported by D’Agostino et al. who demonstrated an increased wound healing and regeneration capacity of healthy HaCaT keratinocytes by H-HA/L-HA in a scratch assay [36]. We were able to confirm this improved wound-healing capacity not only in healthy primary keratinocytes but also after irradiation. 

ADSC-CM is well known for its positive effect on cell regeneration and proliferation. In our study, we found a trend for a higher cell migration with 3-fold concentrated ADSC-CM in the non-irradiated control. Li et al. showed that ADSC-CM improves cellular antioxidant response and induces the expression of procollagen type I synthesis inhibitors in keratinocytes after UVB irradiation [37]. In a further study, a positive effect on the cell migration and viability of human tympanic membrane keratinocytes and on a keratinocyte cell line was demonstrated as well. ADSC-CM stimulated the keratinocyte growth, replication, and expression of wound-healing cytokines [38]. However, and contrary to our expectations, the cell viability experiments revealed a rather inhibiting effect of 3-fold concentrated ADSC-CM. In this study, CM from a human ADSC cell line was used. Patient-dependent individual factors, such as high age, adiposity, comorbidities, or possible treatments could have a negative effect on ADSC’s regenerative potential to grow and differentiate and therefore on their secretome [10,39,40,41,42]. There still is the need for further experiments with ADSCs from different patients to analyze their interindividual differences, their impact on ADSC behavior [43,44], and, in a next step, their influence on wound healing.

Based on the promising results of 1 mg/mL H-HA/L-HA and 100 ng/mL IGF-I on cell viability and the migration of irradiated keratinocytes, these groups were further analyzed by quantitative real-time PCR analysis. One mg/mL H-HA/L-HA induced a significant increase in the expression of MKI67 and IL6 in 2- and 5-Gy-irradiated keratinocytes as well as a tendency towards an increase in TNF expression compared to the control group. Tureson et al. noted that daily fractions of about 1 Gy to the skin resulted in an arrest of cell growth for several weeks and a subsequent acceleration in the repopulation of the basal layer keratinocytes during the following weeks of irradiation [26]. Radiation-induced damage causes keratinocyte DNA injury repair via activation of the p53 pathway and a simultaneous release of inflammatory cytokines because of the formation of free radicals. The main cytokines involved in this reaction are Il-1 and Il-6, TNF-α, and TGF-β [24]. We assume that the repopulation could possibly be accelerated by treatment with 1 mg/mL H-HA/L-HA. The increase in IL6 expression in the 1 mg/mL H-HA/L-HA group could be a sign of a beginning of an increase in the cellular immune response leading to a better recovery process after irradiation in vivo. This would finally lead to faster wound healing and could reduce the severity of radiation dermatitis symptoms.

Nevertheless, the contribution of growth factors, in particular IGF-I and HA, to tumor cell growth needs to be evaluated critically. The IGF-I receptor is overexpressed in many tumor cells. It became apparent that it is involved in certain steps of the metastatic process [45,46]. Furthermore, Matsui et al. have highlighted the possibility of sodium hyaluronate to increase the growth of colon adenocarcinoma cells. Nevertheless, their findings are limited to cancer cells at wound sites with no data about a systemic significance given [47]. However, the oncogenic effects can be balanced and counteracted. This can be achieved by antisense oligodeoxynucleotides or antisense RNA treatment [48,49,50]. After injection of dominant negative mutant IGF-1R, tumor cell apoptosis was initiated, cancer growth was inhibited, and the metastatic proliferation was decreased [51]. Moreover, it must be taken into consideration that, as potential treatment for radiation dermatitis, both IGF-I and HA would be applied topically. It is debatable whether after transdermal application their systemic levels would reach levels high enough to promote tumor growth. 

After irradiation with 5 Gy, we observed an outrank of the migration rate and viability of the collagen-elastin scaffold when comparing the untreated control groups to the group irradiated with 2 Gy. The positive effects of irradiation on the migration and proliferation of keratinocytes were already observed after helium–neon laser irradiation [52]. Similar results were described after irradiation of fibroblasts and ADSCs [53,54]. Goetze et al. postulated a possible modulation of tumor cell migration behavior after irradiation [55]. Additional data highlight the involvement of exosomes in the bystander signaling induced by irradiation. This process can initiate the membrane signaling and functional changes of transfer proteins. A detailed evaluation and interpretation of this effect needs to be addressed in further studies.

Due to the lack of both long-term results and in vivo applicability, which is beyond the scope of this study to evaluate, the generalization of our results is still limited. Within this study, the effect of different treatment options on one cell population was analyzed. However, there are further cell populations in human skin tissue that need to be taken into consideration when evaluating such a novel therapeutic option. The experiments were performed with keratinocytes from two patients. On the one hand, the usage of primary cells simulates the in vivo situation, on the other hand the inclusion of further patients would be necessary for better generalization of the results. 

The study provides a new insight into a possible new and promising therapeutic strategy to counteract the radiation-induced skin changes which could be led back to the positive influence of 1 mg/mL H-HA/L-HA and 100 ng/mL IGF-I on viability, migration, and chemokine induction. The novel findings could finally help to improve current therapeutic strategies for dermal complications after irradiation. Further research is needed to remove these limitations and provide the necessary data to confirm generalizability, long term results, and in vivo applicability. 

## 5. Conclusions

One mg/mL H-HA/L-HA and 100 ng/mL IGF-I were able to mitigate the irradiation damage of primary keratinocytes by stimulating viability and migration and reducing cell apoptosis and necrosis. These findings indicate that the negative effects of irradiation on keratinocytes inside the patient’s skin could be decreased and possibly even counterbalanced with HA and IGF-I treatment. It can be concluded that both 1 mg/mL HA/L-HA and 100 ng/mL IGF-I may offer a promising therapeutic option for the prevention and treatment of irradiation dermatitis. 

## Figures and Tables

**Table 1:** Experimental groups for the 2D culture.

Group	Supplement	Concentration
1 (Control)		
2	H-L-HA (50/50)	1 mg/mL
3	H-L-HA (50/50)	0.15 mg/mL
4	L-HA	1 mg/mL
5	L-HA	0.15 mg/mL
6	H-HA	1 mg/mL
7	H-HA	0.15 mg/mL
8	IGF-I	100 ng/mL
9	KGF	100 ng/mL
10	PL	50 µL/mL
11	ADSC Control CM	3-fold concentrated
12	ADSC CM	3-fold concentrated

IGF-I = insulin-like growth factor 1, KGF = keratinocyte growth factor, PL = platelet lysate, ADSC CM = ADSC conditioned medium, ADSC CCM = ADSC conditioned control medium, H-HA = high molecular weight hyaluronic acid, L-HA = low molecular weight hyaluronic acid.

**Table 2:** qPCR primer sequences.

Gene	5′-3′ Primer Sequence
*GAPDH*	For: ACATCAAGAAGGTGGTGAAGCAGG Rev: ACAAAGTGGTCGTTGAGGGCAA
*P53*	For: GAAAACCTACCAGGGCAGCT Rev: GGGAGTACGTGCAAGTCACA
*TNF*	For: TGGGATCATTGCCCTGTGAG Rev: GGTGTCTGAAGGAGGGGGTA
*TGFb1*	For: CATGGAGGACCTGGATGCC Rev: TCCTGAAGACTCCCCAGACC
*IL6*	For: AAAGAGGCACTGGCAGAAAA Rev: TTTCACCAGGCAAGTCTCCT
*IL8*	For: GTTCCACTGTGCCTTGGTTT Rev: GCTTCCACATGTCCTCACAA
*IL1B*	For: GCTCGCCAGTGAAATGATGG Rev: GGTGGTCGGAGATTCGTAGC
*COL1A1*	For: GCTCTTGCAACATCTCCCCT Rev: CCTTCCTGACTCTCCTCCGA
*MKI67*	For: TCGACCCTACAGAGTGCTCA Rev: GTGGGGAGCAGAGGTTCTTC
*PTGS-2/COX-2*	For: ATGGAAACAGAGAAGTTGGCAG Rev: GATACAGCTCCACAGCATCG
*FN1*	For: GAGAAGTATGTGCATGGTGTCAG Rev: AATACTTCGACAGGACCACTTGA

## Data Availability

Data available on reasonable request.
